# Supplementary material for: A simple method to measure methane emissions from indoor gas leaks
Source: PLoS One. 2023 Nov 30;18(11):e0295055. doi: 10.1371/journal.pone.0295055 (PMC10688665; doi:10.1371/journal.pone.0295055)
Supplement: S1 Appendix — (PDF) [file pone.0295055.s001.pdf]

S1 Appendix: Selected Building Locations and Characteristics

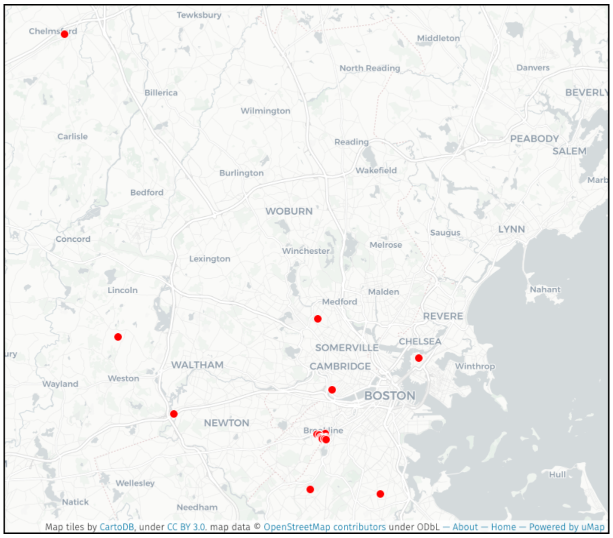

Figure 1. Map of locations of buildings studied

Table 1. General locations of buildings studied

**City or Town Location**	**Buildings**
Boston (Dorchester neighborhood)	1
Boston (Roslindale neighborhood)	1
Brookline	10
Cambridge (Cambridgeport neighborhood)	1
Chelmsford	1
East Boston	1
Newton (Auburndale neighborhood)	1
Somerville (West Somerville)	1
Weston	1

Table 2. General characteristics of the buildings in the study

**Experiment location name**	**Single-family or multi-family home**	**Building occupant(s) are owners or renters**	**Number of families in studied building**	**Home age (years)**	**Approximate subterranean level of basement (%)**	**Basement empty volume (V_{empty}) (ft^3)**	**Basement adjusted volume (V_{adj}) (ft^3)**
R1	single	Owners	1	96	37.5	4559	4418
R2	single	Owners	1	202	85	2388	2184
R3	single	(See R4)	(See R4)	(See R4)	(attic)	814	702
R4	single	Owners	1	17	90	1110	1083
R5	single	Owners	1	63	85	(Basement air CH_4 concentration below threshold - experiment not performed)	
R6	multi	Both	2	122	80	8399	8283
R7	multi	(see R6)	(see R6)	(see R6)	(see R6)	2256	2169
R8	single	Owners	1	102	80	4282	3931
R9	single	Owners	1	112	85	6787	5707
R10	multi	Both	2	152	85	6452	5830
R11	multi	Both	2 in left side (in study) plus 2 in right side (not in study)	38	80	4472	4274

R12	single	Owners	1	102	85	3653	3517
R13	single	Owners	1	96	75	3758	3234
R14	single	Renters	1	67	25	(Basement air CH_4 concentration below threshold - experiment not performed)	
R15	multi	Both	3	102	50	8549	7842
R16	single	Owners	1	93	85	3674	3471
R17	multi	Both	3	109	80	6933	5764
R18	multi	Both	2, left side only	128	80	5220	4669
R19	single	Owners	1	171	72.5	2971	2829
R20	single	Owners	1	122	80	4215	3835
R21	single	Owners	1	122	50	3606	3225
R22	single	Owners	1	140	80	(Basement air CH_4 concentration below threshold - experiment not performed)	
