# Supplementary material for: A simple method to measure methane emissions from indoor gas leaks
Source: PLoS One. 2023 Nov 30;18(11):e0295055. doi: 10.1371/journal.pone.0295055 (PMC10688665; doi:10.1371/journal.pone.0295055)
Supplement: S2 Appendix — (PDF) [file pone.0295055.s002.pdf]

S2 Appendix: Ambient CH_4 air concentration measurements

S2 Appendix section 1: Differences between ambient outdoor and basement air CH_4 concentrations

Ambient outdoor and basement air CH_4 concentrations were measured and used to determine whether to proceed with RCM and the bag method. The differences between the outdoor and basement measurements for each experiment location are shown in figure 2. Mean outdoor and basement ambient air CH_4 concentrations were 2.05 ppmv and 3.67 ppmv respectively, with medians 2.04 ppmv and 3.27 ppmv respectively (n=22). Differences between outdoor and basement air CH_4 concentrations ranged between 0-4.3 ppmv (mean 1.6 ppmv, median 1.2 ppmv).

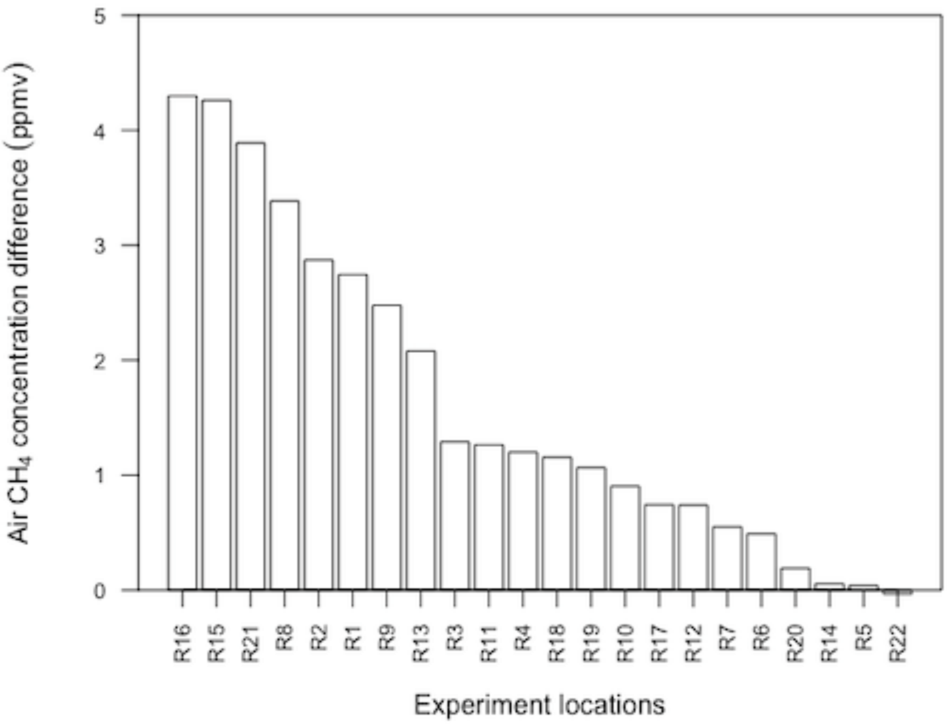

Figure 2. Differences between outdoor air CH_4 concentrations and basement air CH_4 concentrations.

S2 Appendix section 2: Ambient air CH_4 concentrations by floor for single-family and multi-family homes

Multiple air CH_4 concentration readings were measured throughout each floor of each building studied prior to performing RCM or the bag method. Figure 3 shows the minimum, maximum, mean and median of these average measurements by floor and home type. Across all floors, multi-family homes had both a higher average minimum indoor air CH_4 concentration compared to single-family homes (2.4 ppmv versus 2.05 ppmv) and a higher average maximum indoor air CH_4 concentration compared to single-family homes (6.8 ppmv versus 3.8 ppmv).

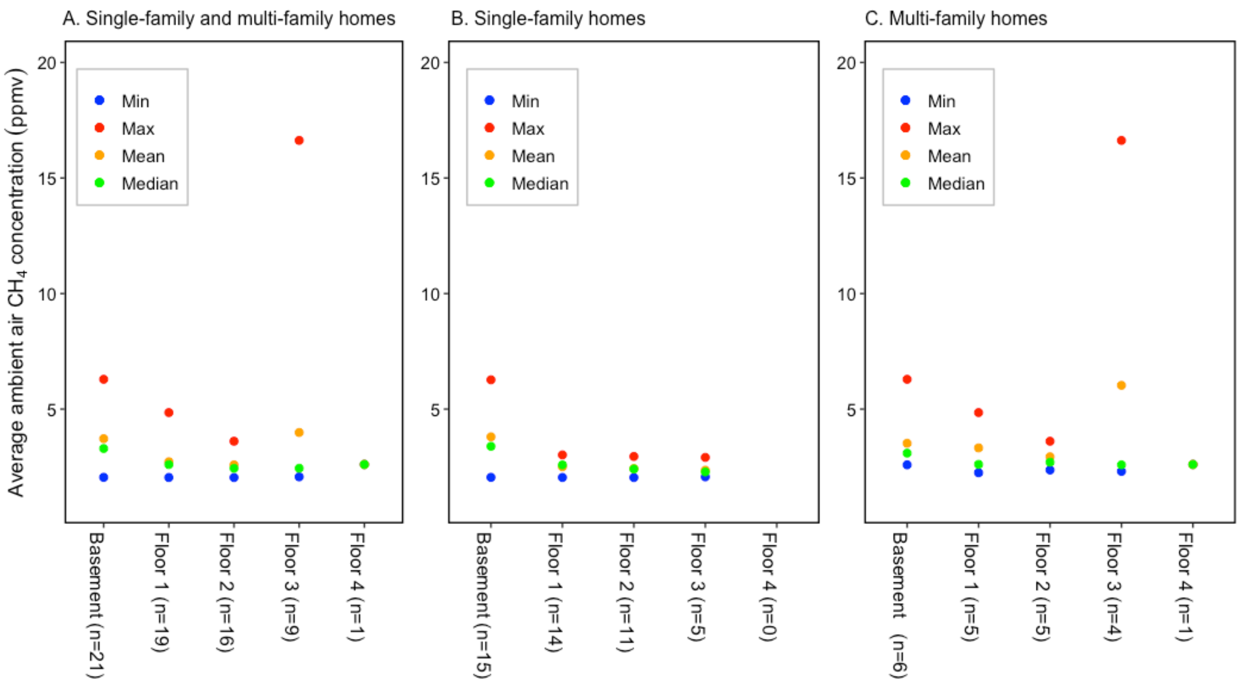

Figure 3. Average air CH_4 concentrations by floor for single and multi-family homes
