# Supplementary material for: A simple method to measure methane emissions from indoor gas leaks
Source: PLoS One. 2023 Nov 30;18(11):e0295055. doi: 10.1371/journal.pone.0295055 (PMC10688665; doi:10.1371/journal.pone.0295055)
Supplement: S3 Appendix — (PDF) [file pone.0295055.s003.pdf]

S3 Appendix: Air CH_4 concentrations over time during air flushing phase

Figure 4A shows air CH_4 concentrations over time during RCM flushing (flushing data unavailable for experiment location R3 - an attic space - because the GasScouter was in use in the basement of the same home). Figure 4B shows air concentrations over time during the bag method flushing. RCM flushing used more fans with higher volume air flow compared to the bag method which shows more high frequency air CH_4 concentrations variations. However, both methods were able to achieve a flushed steady state air CH4 concentration in the room chamber. Dashed lines in figures 4A and 4B indicate smoothed fits to highlight overall trends.

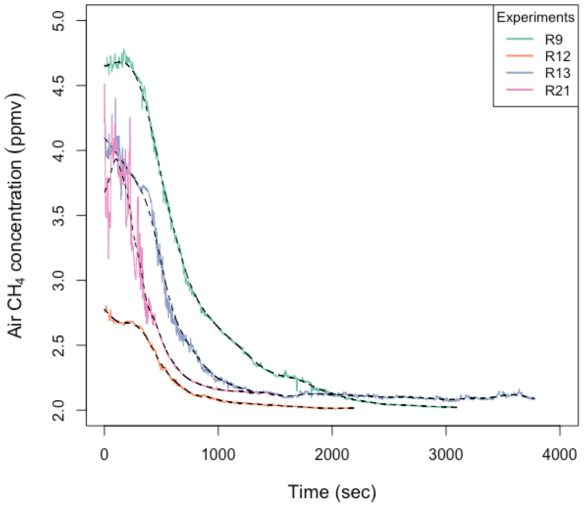

Figure 4A. Air CH_4 concentrations during room chamber method (RCM) flushing

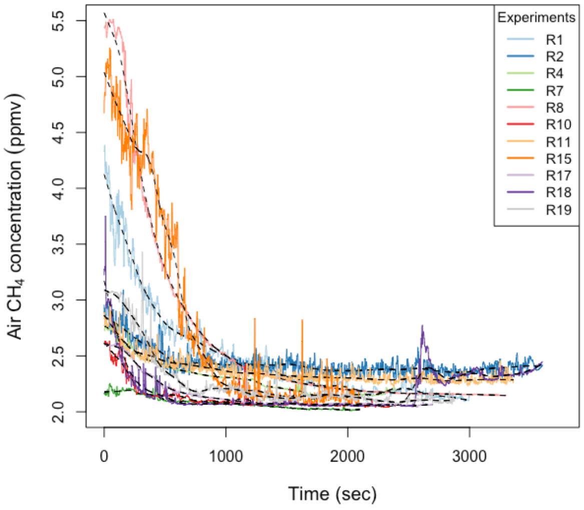

Figure 4B. Air CH_4 concentrations during bag method flushing
