# Supplementary material for: A simple method to measure methane emissions from indoor gas leaks
Source: PLoS One. 2023 Nov 30;18(11):e0295055. doi: 10.1371/journal.pone.0295055 (PMC10688665; doi:10.1371/journal.pone.0295055)
Supplement: S4 Appendix — (PDF) [file pone.0295055.s004.pdf]

S4 Appendix: Room Chamber Method (RCM)

S4 Appendix section 1: Room volume measurements

For each RCM experiment, an empty basement volume (V_{empty}) was calculated, together with the volume consumed by objects stored in the basement ($V_{objects}$). Objects' volumes ranged between 2.4-16.9% of empty basement volumes, with mean 8.5% and median 8.4%. This information was later used to set a standard room chamber volume adjustment factor for bag method calculations. Figure 5 shows $V_{objects}$ as a percentage of V_{empty} for each RCM experiment.

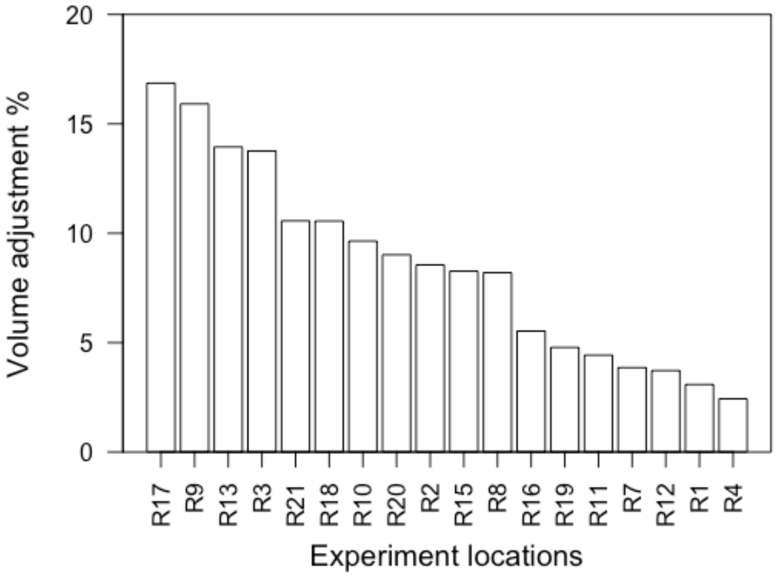

Figure 5. Volumes of objects ($V_{objects}$) divided by the empty basement volumes (V_{empty})

S4 Appendix section 2: Example of calculating rate of change of air CH_4 concentration using "simple slope"

Using experiment location R8 as an example, with a timespan of 600 sec between air CH_4 concentration sample points, and an adjusted room chamber volume of 3931 ft^2, RCM daily emissions are calculated using the "simple slope" calculation method as follows:

$$[\ (CH_4 \text{ at } t_n - CH_4 \text{ at } t_0)\ /\ n\ ]\ \times 60 \times 60 \times 24 \times\ V_{adj}\ \times 10^{-6}$$

$$= [\ (2.382 - 2.173)\ /\ 600\ ]\ \times 60 \times 60 \times 24 \times\ 3931 \times 10^{-6}$$

$$= 0.118\ ft^3\ day^{-1}$$ (the corresponding RCM fitted tangent estimate was 0.1131 ft^3 day^{-1})

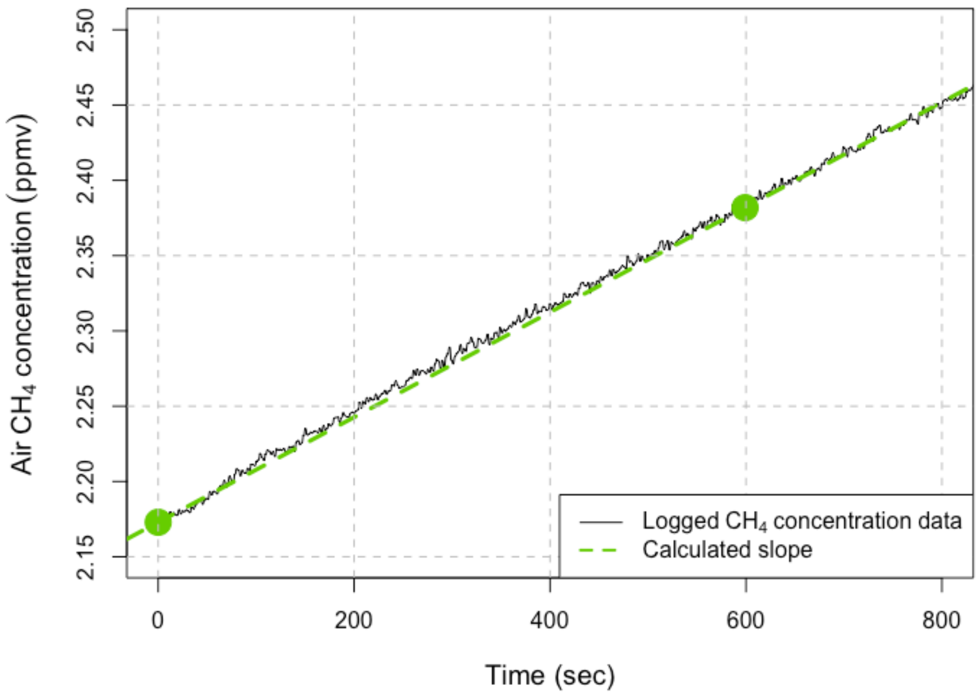

Figure 6. Example of room chamber method (RCM) emissions calculation using "simple slope" (green points represent air CH_4 concentration samples 600 sec apart, experiment location R8)

S4 Appendix section 3: Example of calculating rate of change of air CH4 concentration using "fitted tangent"

Air CH_4 concentration over time in a room chamber can be modeled using $CH_4(t) = S(1 - e^{-kt})$ where t is time, S is the steady state air CH_4 concentration in the room chamber, -k is a time constant. Modeling was done using functions lm() and nls() from the R Stats Package, 'stats', version 3.6.2. In practice, we found that nonlinear weighted least squares regression resulted in a lower sum of least squares residuals when using $CH_4(t) = S - \alpha e^{-kt}$. The rate of change of air CH_4 concentration is given by the first derivative (i.e. curve tangent) of this equation and is $dCH_4/dt = k\alpha e^{-kt}$ which at t=0 is $k\alpha$ and is the maximum rate of change in air CH_4 concentration. For modeling, nonlinear weighted least squares regression weights were configured to ensure the modeled fit passed through the first data point. Using experiment location R18 as an example, after data modeling, $CH_4(t) = 4.2 - 1.75e^{-0.00026t}$ and $dCH_4/dt = 0.000445$ CH_4 ppmv sec^{-1}. In this example $V_{adj} = 4669$ ft^3, resulting in daily CH_4 emissions of 0.000445 x 60 x 60 x 24 x 4669 x 10^{-6} = 0.179 ft^3 day^{-1} (the corresponding RCM simple slope estimate was 0.198 ft^3 day^{-1}).

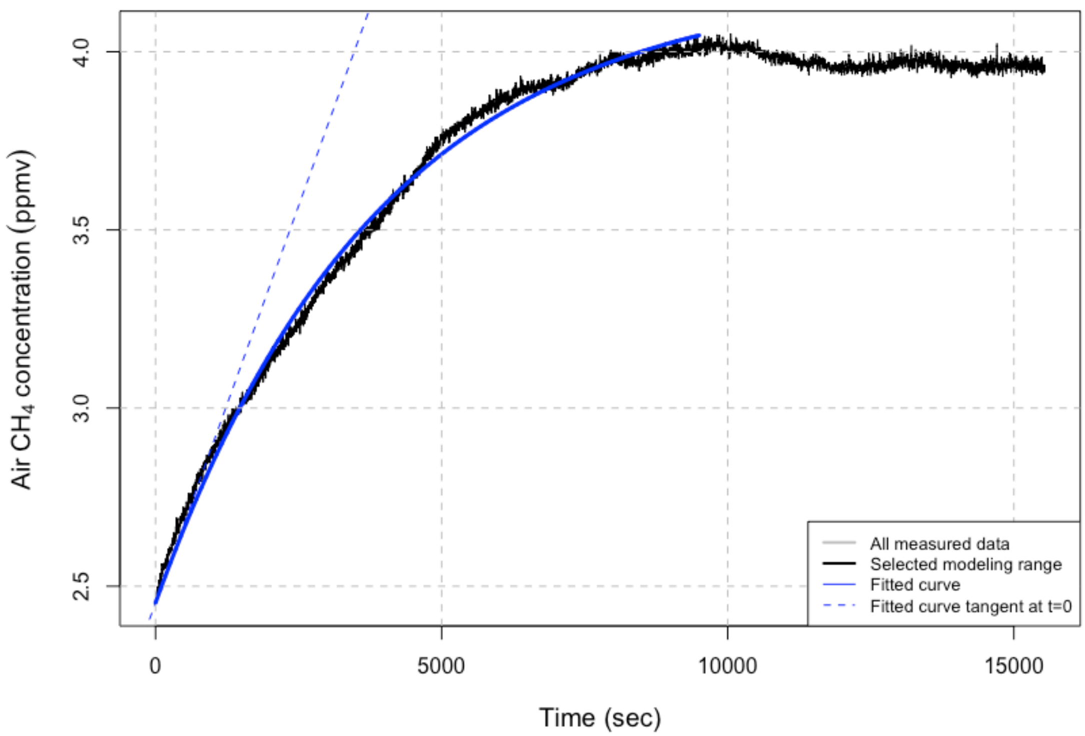

Figure 7. Example of room chamber method (RCM) emissions calculation using “fitted tangent” (experiment location R18)

S4 Appendix section 4: Effects on calculated emissions of varying timespan used in calculation methods

Figure 8A shows the effect on calculated daily emissions of varying the timespan used in the simple slope calculation method, on an RCM control test (R8_RCM_9). The actual test flux is shown in orange. Similarly, figure 8B shows the effect of increasing the timespan used in the fitted tangent calculation method. Figures 9A and 9B show the effect of varying timespan on calculated emissions for all experiments, with timespan increments of 300 and 2000 sec respectively. Fitted tangent calculation tests ranges from t=1500 to t=22000 (R3 lacked sufficient data to perform the test for fitted tangent modeling).

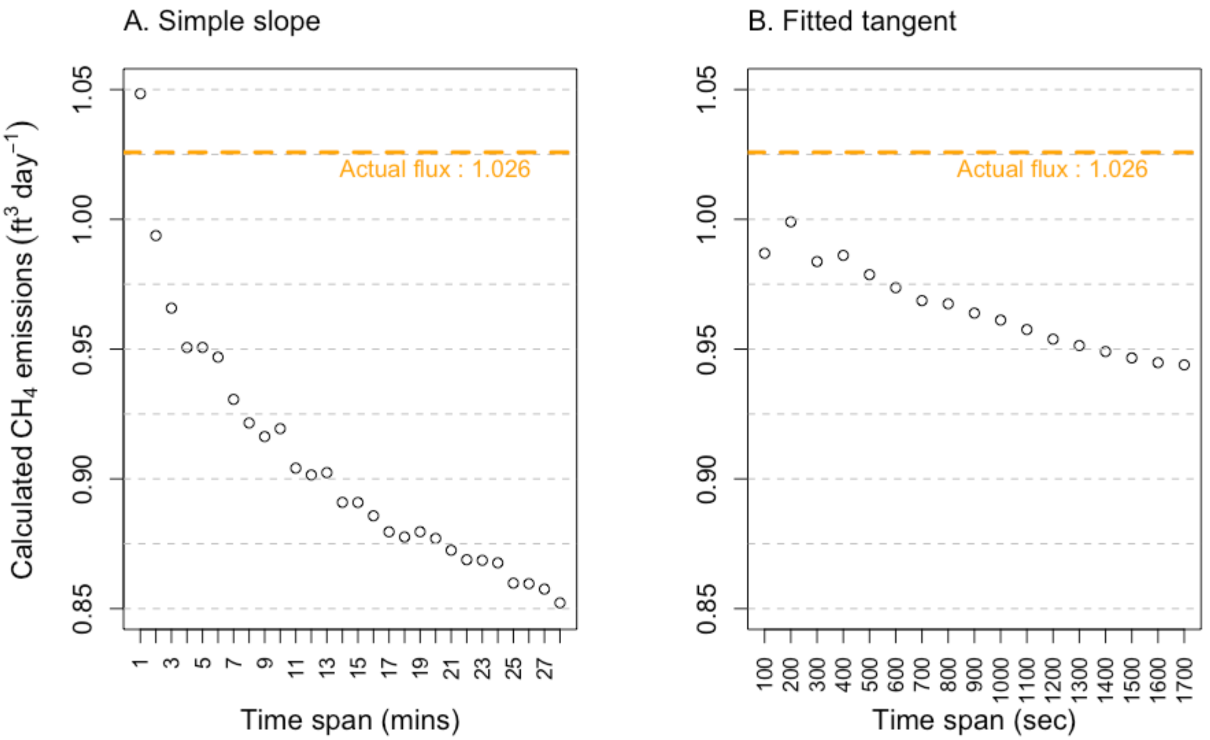

Figure 8. Testing the effect of varying timespan on calculated emissions for a control test R8_RCM_9

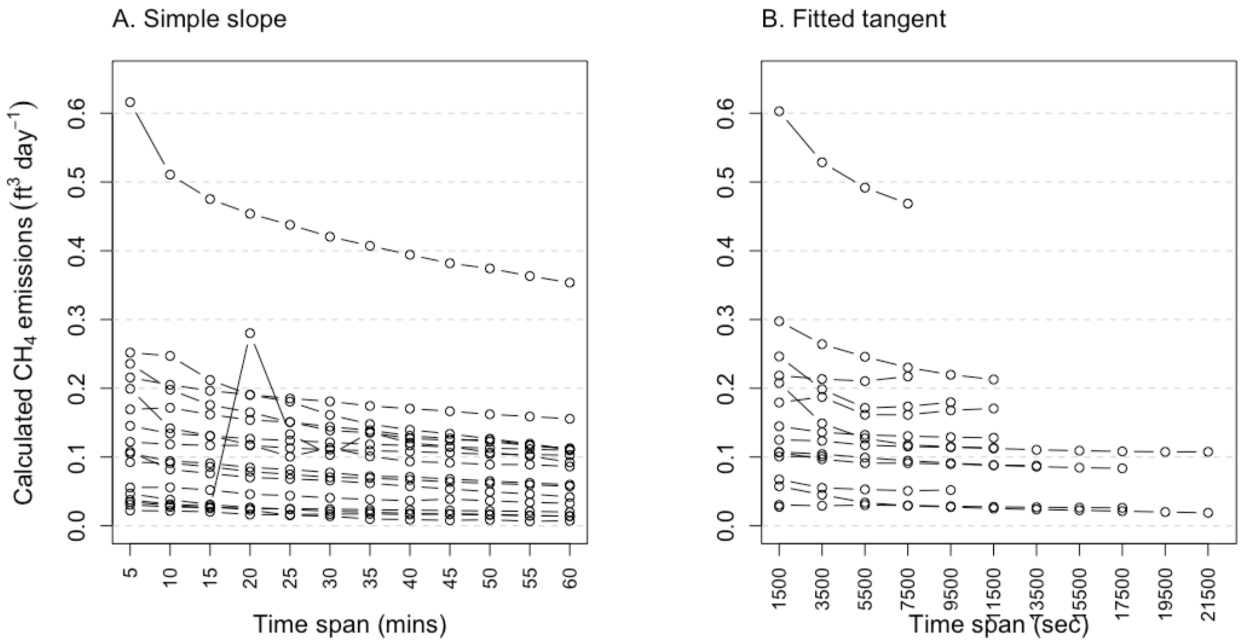

Figure 9. Line charts showing the effect of varying timespan on calculated emissions for all experiments.

S4 Appendix section 5: Relationship between unflushed basement ambient CH_4 and calculated emissions

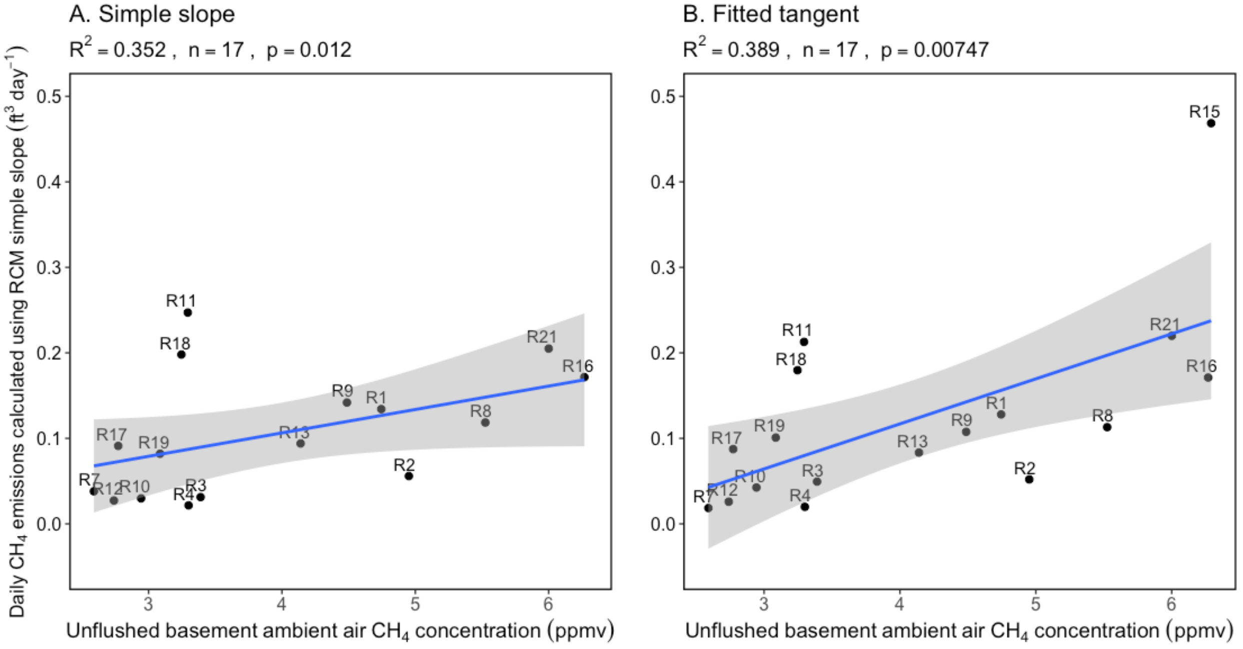

Figure 10. Unflushed basement ambient air CH_4 concentration relationship to daily emissions calculated with simple slope and fitted tangent calculation methods (gray bands indicate 95% confidence intervals)

S4 Appendix section 6: Examples of air CH_4 concentration spikes

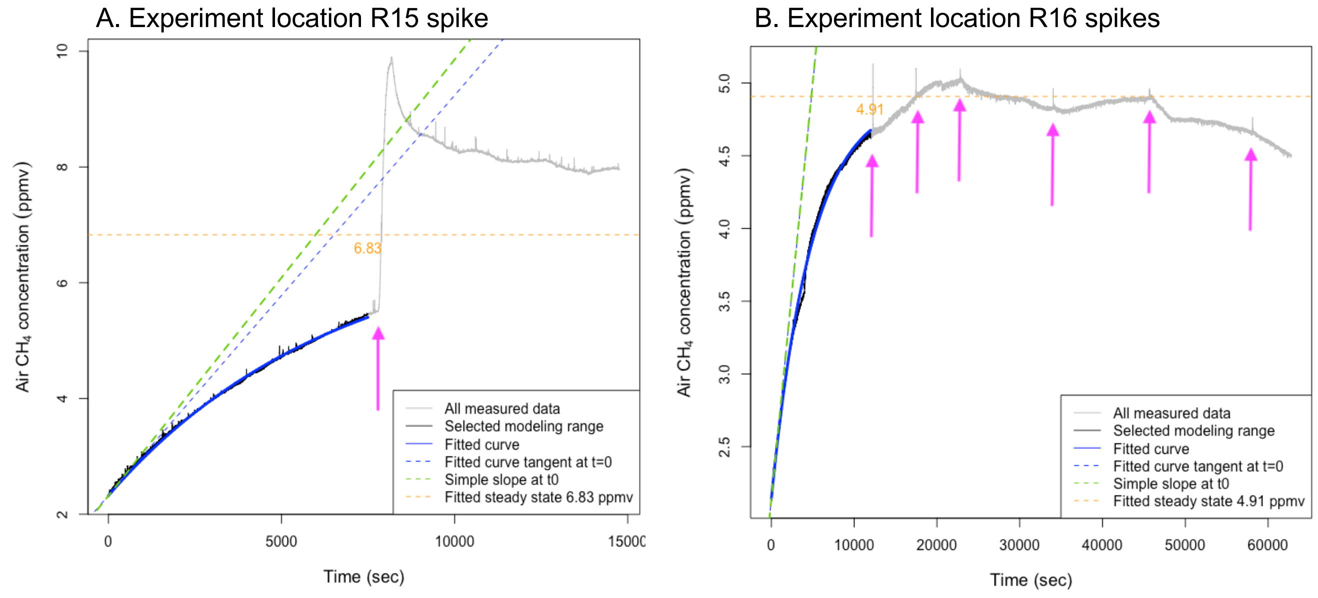

Figure 11. Examples of air CH_4 concentration spikes (pink arrows) coinciding with unburned methane possibly from NG domestic water heater cycling ("fuel slip") (experiments R15 and R16)

S4 Appendix section 7: Examples of fluctuations in air CH_4 concentrations after rise phase

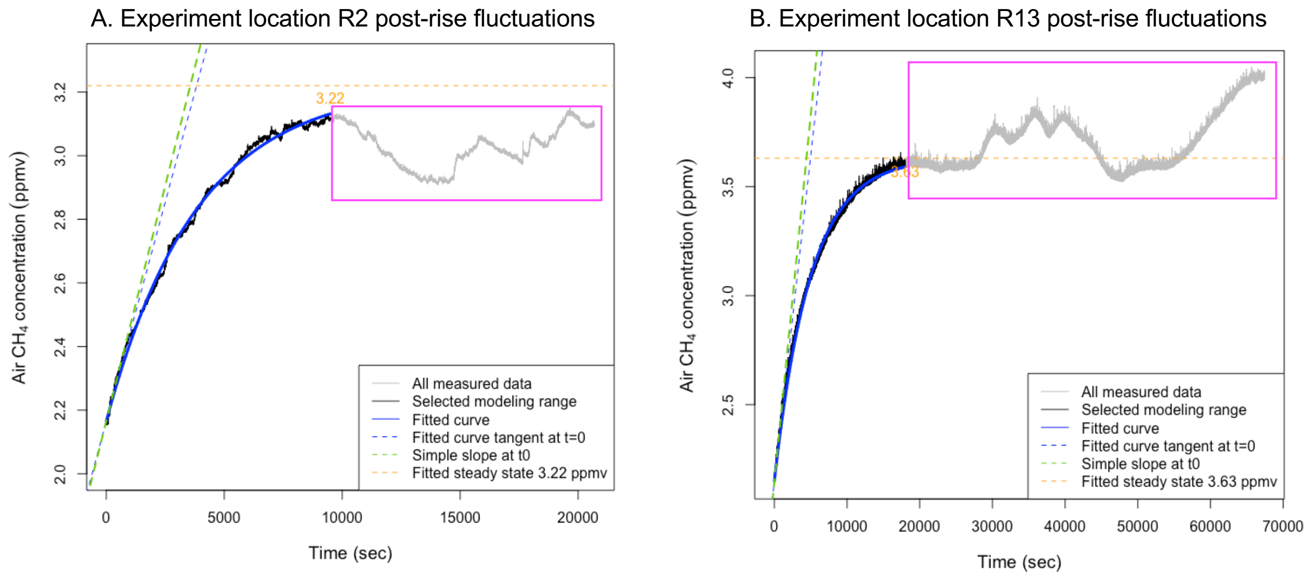

Figure 12. Examples of post-rise air CH_4 concentration fluctuations (pink boxes) (experiment locations R2 & R13)

S4 Appendix section 8: Example of poor air mixing and remedy

Example of an experiment with poor air mixing resulting in reading fluctuations (figure 13A) and results after addressing the issue (figure 13B) by excluding spaces and sub-rooms in the basement that didn't contain NG infrastructure but were making air circulation challenging.

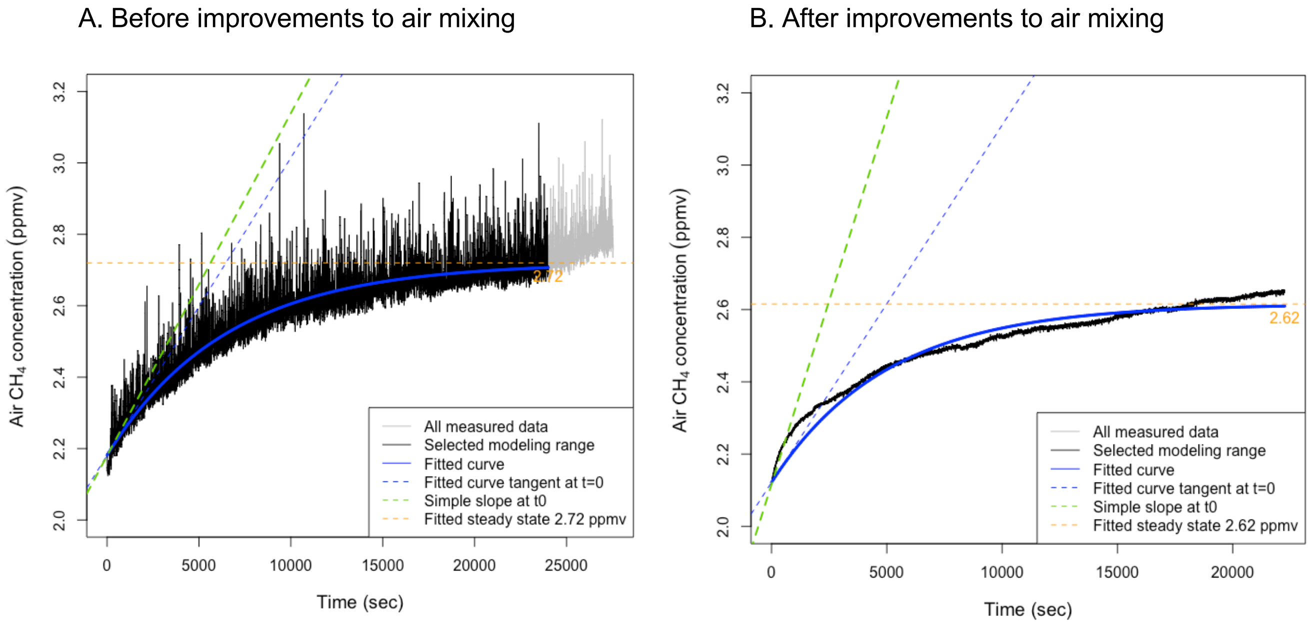

Figure 13. Example of experiment with poor air mixing resulting in reading fluctuations (A) and results after addressing the issue (B) (experiment locations R6 and R7 respectively).

S4 Appendix section 9: Pressure and air CH4 concentration over time

A multi-day experiment was performed from November 23 2022 to November 25 2022 to explore how indoor and outdoor atmospheric pressure may vary with indoor air CH_4 concentrations. The experiment was performed in the same closed basement in which the majority of metered control tests had been performed. During the experiment, the home was vacant and all gas appliances were turned off. Four 4960 CFM (140 m^3min^{-1}) 20 inch (51 cm) floor fans (Pelonis model PFE50A4ABB) were left on to circulate the air in the basement. A differential manometer (Omega HHP886U) measured indoor and outdoor pressure every 1 second. The outdoor pressure input was attached to a tube exiting the basement through a sealed hole in a window frame. The GasScouter was located in the same location as in metered control tests. There was no apparent relationship between pressures and air CH4 concentration. It is possible that fluctuations in air CH_4 concentrations in this scenario could be due to pressure fluctuations in the NG distribution system. Figure 14 shows measured fluctuations in air CH_4 concentration (black), indoor and outdoor atmospheric pressures (blue and red, with solid lines showing smoothed fits) and the calculated difference between the smoothed pressure fits (green).

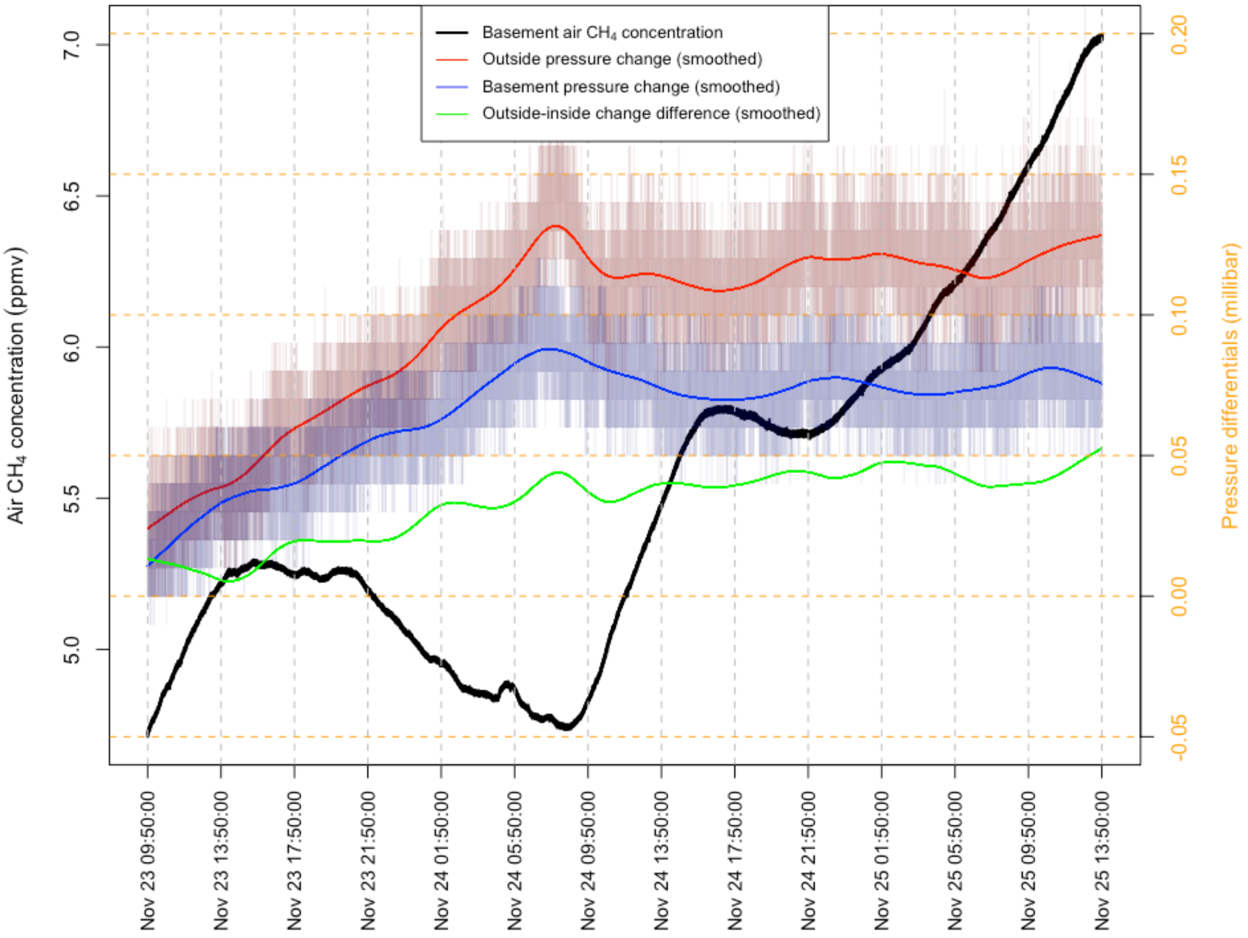

Figure 14. Air pressures and air CH_4 concentration
