# Supplementary material for: A simple method to measure methane emissions from indoor gas leaks
Source: PLoS One. 2023 Nov 30;18(11):e0295055. doi: 10.1371/journal.pone.0295055 (PMC10688665; doi:10.1371/journal.pone.0295055)
Supplement: S5 Appendix — (PDF) [file pone.0295055.s005.pdf]

S5 Appendix: Simplified Room Chamber Method ("Bag method")

S5 Appendix section 1: Air sampling kit procedure

The air sampling kit consisted of two 0.5 L Tedlar bags with polypropylene combo valves (Restek 22049) and a gas sampling bulb (Heathrow Scientific 56HV89). The bulb can be used to create either suction or inflation depending on which side of the bulb is connected. To evacuate a bag before sample, the suction side of the bulb was connected tightly with a plastic tube to the bag's valve nipple. The bag valve was opened and the bulb was pumped by hand until the bag was completely evacuated. The bag valve was then closed and the bulb removed. When filling the bag, the unconnected bulb was first pumped 10 times to flush it. The inflation side of the bulb was connected tightly with a plastic tube to the bag's valve nipple. The bag valve was opened and the bulb was pumped by hand until the bag was filled. The bag valve was then closed and the bulb removed

S5 Appendix section 2: Testing convergence of RCM and the bag method

The relative differences between calculated emissions and emission size are shown in figure 15. As emission sizes increase, the difference between the two methods decreases with method relative estimates diverging below 0.2 ft^3 day^{-1}. These results suggest that we have reached the lower limits of flux size estimation ability using this overall methodological approach, but also that we expect this methodological approach to resolve larger fluxes than tested with increasing precision.

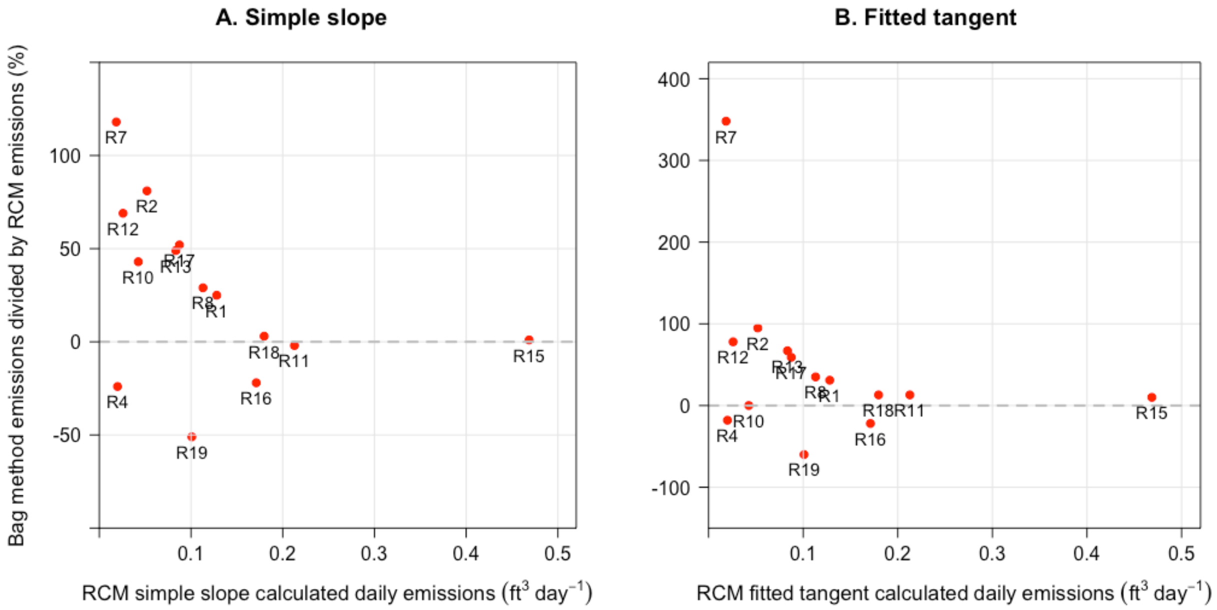

Figure 15. Method convergence testing (red dots represent individual experiments).
