# Supplementary material for: A simple method to measure methane emissions from indoor gas leaks
Source: PLoS One. 2023 Nov 30;18(11):e0295055. doi: 10.1371/journal.pone.0295055 (PMC10688665; doi:10.1371/journal.pone.0295055)
Supplement: S6 Appendix — (PDF) [file pone.0295055.s006.pdf]

S6 Appendix: Testing methods with metered CH_4 releases

S6 Appendix section 1: RCM metered control CH_4 flux tests

Different combinations of 2 test gasses (100% CH_4 and 2.5% CH_4) and flow rates were used in 2 of the authors' basements to test RCM against 3 orders of magnitude of daily fluxes. All test gas equipment was located outdoors. Test gas flow rates were controlled using a regulator and an inline needle valve to provide fine control. To measure flow rates, we used a Dwyer RMA-13 general use flow meter. The outlet of the flow meter was connected to a 25 ft tube fed into the test basement through a sealed hole in a window frame. A test was done to ensure flow rate at both ends of the tube was the same and unimpeded. The end of the tube was positioned in one corner of the basement. The GasScouter was placed in approximately the center of the basement away from any gas appliances. Exterior windows and doors were opened and basement air was flushed out and replaced with outdoor air using 4 adjustable speed tilting head floor fans (4960 CFM (140 $m^3 min^{-1}$) 20 inch (51 cm); Pelonis model PFE50A4ABB) and two-three box fans (20 inch (51 cm) 1800 CFM (51 $m^3 min^{-1}$); Lasko model 3733). Air flushing continued until a flushed steady state was observed using the GasScouter and air CH_4 concentrations were approximately steady and extremely close to outdoor ambient air CH_4 concentrations (which were measured before tests). Exterior windows and doors were closed and fans reoriented to provide maximum air CH_4 mixing and circulation in the basement. The test gas was turned on and flow rate set and carefully monitored to keep it constant. Basement air CH_4 concentration was monitored for safety in the basement throughout all tests and we ensured that concentrations were never more than an order of magnitude less than the CH_4 lower explosive limit. Air CH_4 concentrations were logged during the tests and fluxes calculated using both calculation simple slope and fitted tangent methods. Some potential error exists in control equipment such as flow meter, regulators, test gasses and basement volume calculations. Tests were performed in 2 different basements from experiment locations R8 (13 tests) and R16 (1 test). We adjusted flow rates to account for the specific gravity of CH_4 relative to air:

$$F_{adj} = F \times C \times SG_{CH4}^{-½} \text{ L min}^{-1} \qquad \text{(i) for 100\% } CH_4 \text{ test gas}$$

$$F_{adj} = F \times (C \times SG_{CH4}^{-½} + (1-C) \times SG_{air}^{-½}) \text{ L min}^{-1} \qquad \text{(ii) for 2.5\% } CH_4 \text{ test gas}$$

where F_{adj} is the adjusted flow rate in liters per minute, F is measured flow rate, C is the percent of CH_4 in the test gas, $SG_{methane}$ is the specific gravity of CH_4 (0.5537) and SG_{air} is the specific gravity of air comprising the test gas balance (1). Control fluxes were calculated using :

$$\text{Daily flux} = F_{adj} \times C \times 60 * 24 / 28.32 \text{ ft}^3 \text{ day}^{-1}$$

Table 3 and figure 16 shows test configurations and results. Unless noted otherwise, all NG leakage in the basement was removed by switching off the NG supply in the test basement before testing. In cases where the NG supply was not switched off, the RCM calculated control

fluxes were adjusted by removing leak fluxes calculated in prior RCM experiments in this basement. Because in these cases the background leak rates were typically at least an order of magnitude less than the control test fluxes, we feel confident in the quality control tests and note that this confidence may be increased by studying a wider range of room types, sizes and different flux rates.

Table 3. RCM metered CH_4 flux tests results

Control test	Test gas (% CH_4)	Control test flux ($ft^3\ day^{-1}$)	Calculated flux using simple slope ($ft^3\ day^{-1}$)	Calculated flux using fitted tangent ($ft^3\ day^{-1}$)
R8_RCM_1	2.5	0.19	0.18	0.19
R8_RCM_2	2.5	0.19	0.20	0.20
R8_RCM_3	2.5	0.26	0.19	0.19
R8_RCM_4	2.5	0.26	0.20	0.21
R8_RCM_5	2.5	0.38	0.31	0.32
R8_RCM_6	2.5	0.51	0.44	0.46
R8_RCM_7	2.5	0.64	0.57	0.60
R8_RCM_8	2.5	0.77	0.66	0.69
R8_RCM_9	2.5	1.03	0.92	0.94
R8_RCM_10	2.5	5.00	4.27 (4.39) (3)	5.04 (5.16) (3)
R8_RCM_11	100	54.67	54.52 (54.64) (3)	59.08 (59.20) (3)
R8_RCM_12	100	61.51	61.70 (61.82) (1,3)	65.92 (66.00) (3)
R8_RCM_13	100	198.19	192.82 (192.94) (2,3)	201.45 (201.56) (3)
R16_RCM_1	2.5	4.49	3.37 (3.55) (3)	3.71 (3.88) (3)

(1) Test data limited to 320 sec (2) Test data limited to 120 sec (3) Adjusted to account for active NG leak fluxes with pre-adjusted fluxes shown in parentheses

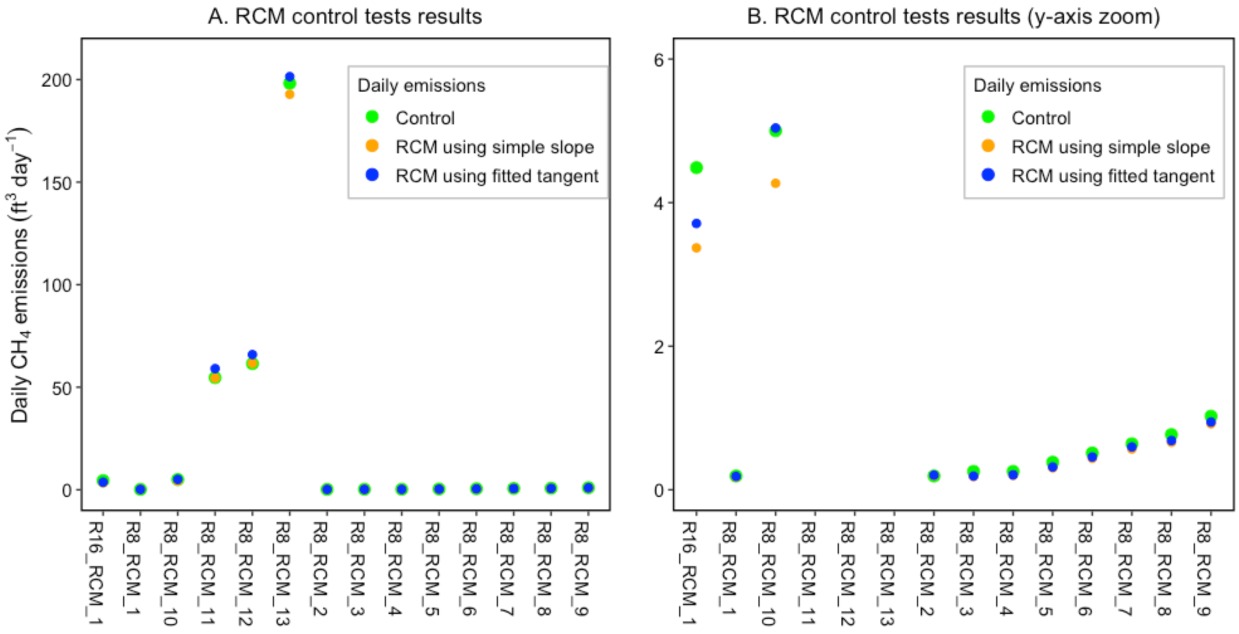

Figure 16. Room chamber method (RCM) control test results, with panel B showing a y-axis zoom.

S6 Appendix section 2: Bag method metered control CH_4 flux tests

The bag method was tested against metered control CH_4 fluxes in 1 author's basement. We were able to test fluxes typical of those we were observing in RCM experiments. The test basement was the same as used for the majority of the RCM control tests and any existing leakage was first eliminated by turning off the NG supply during testing. All test gas equipment was located outdoors. Test gas flow rates were controlled using a regulator and an inline needle valve to provide fine control. To measure flow rates, we used a Dwyer RMA-13 general use flow meter. The outlet of the flow meter was connected to a 25 ft tube fed into the test basement through a sealed hole in a window frame. A test was done to ensure flow rate at both ends of the tube was the same and unimpeded. The end of the tube was positioned in one corner of the basement, the same location used in RCM tests. The GasScouter was placed in the same location used for RCM tests and used for monitoring. Exterior windows and doors were opened and basement air was flushed out and replaced with outdoor air using 2-3 box fans (20 inch (51 cm) 1800 CFM (51 m^3min^{-1}); Lasko model 3733). Air flushing continued until a flushed steady state was observed using the GasScouter and air CH_4 concentrations were approximately steady. Exterior windows and doors were closed and fans reoriented to provide maximum air CH_4 mixing and circulation in the basement. Basement air CH_4 concentration was monitored for safety in the basement throughout all tests and we ensured that concentrations were never more than an order of magnitude less than the CH_4 lower explosive limit. Both Tedlar bags were fully evacuated and the first air sample was taken in one bag (SI 5.1). The test gas was turned on and flow rate set and carefully monitored to keep it constant. The second air sample was taken a recorded amount of time from the beginning of the air CH_4 concentration rise phase as observed on the GasScouter. Air samples were taken at the same location as the GasScouter intake as used in RCM metered control tests. Air samples were later analyzed using the

GasScouter (8 tests) or the G2311-f (2 tests). Control fluxes were calculated in the same way as those used in RCM control testing. Some potential error exists in control equipment such as flow meter, regulators, test gasses and basement volume calculations. Tests were performed in the basement from experiment location R8 (10 tests). We adjusted flow rates to account for the specific gravity of CH_4 as in RCM testing. Table 4 and figure 17 shows test configurations and results. We are confident in the quality control tests and that this sampling method will work with higher magnitude fluxes, while noting that confidence may be increased by studying a wider range of room types, sizes and fluxes.

Table 4. Bag method metered CH_4 flux tests results, where adjusted volume (V_{adj}) is the volume of the room chamber adjusted by removing the volume of objects therein, and the simplified volume (V_{sim}) is the volume of the room chamber multiplied by the average volume consumed by objects in basements measured across this study (f=0.92)

Control test name	Test gas (CH4 %)	Control test flux ($ft^3\ day^{-1}$)	Calculated flux using V_{adj} ($ft^3\ day^{-1}$)	Calculated flux using V_{sim} ($ft^3\ day^{-1}$)
R8_SRCM_1	2.5	0.19	0.13	0.13
R8_SRCM_2	2.5	0.64	0.57	0.56
R8_SRCM_3	2.5	0.26	0.23	0.23
R8_SRCM_4	2.5	0.38	0.33	0.33
R8_SRCM_5	2.5	0.77	0.67	0.66
R8_SRCM_6	2.5	0.26	0.19	0.19
R8_SRCM_7	2.5	1.03	0.91	0.90
R8_SRCM_8	2.5	0.51	0.49	0.48
R8_SRCM_9	2.5	0.38	0.34	0.33
R8_SRCM_10	2.5	0.38	0.38	0.37

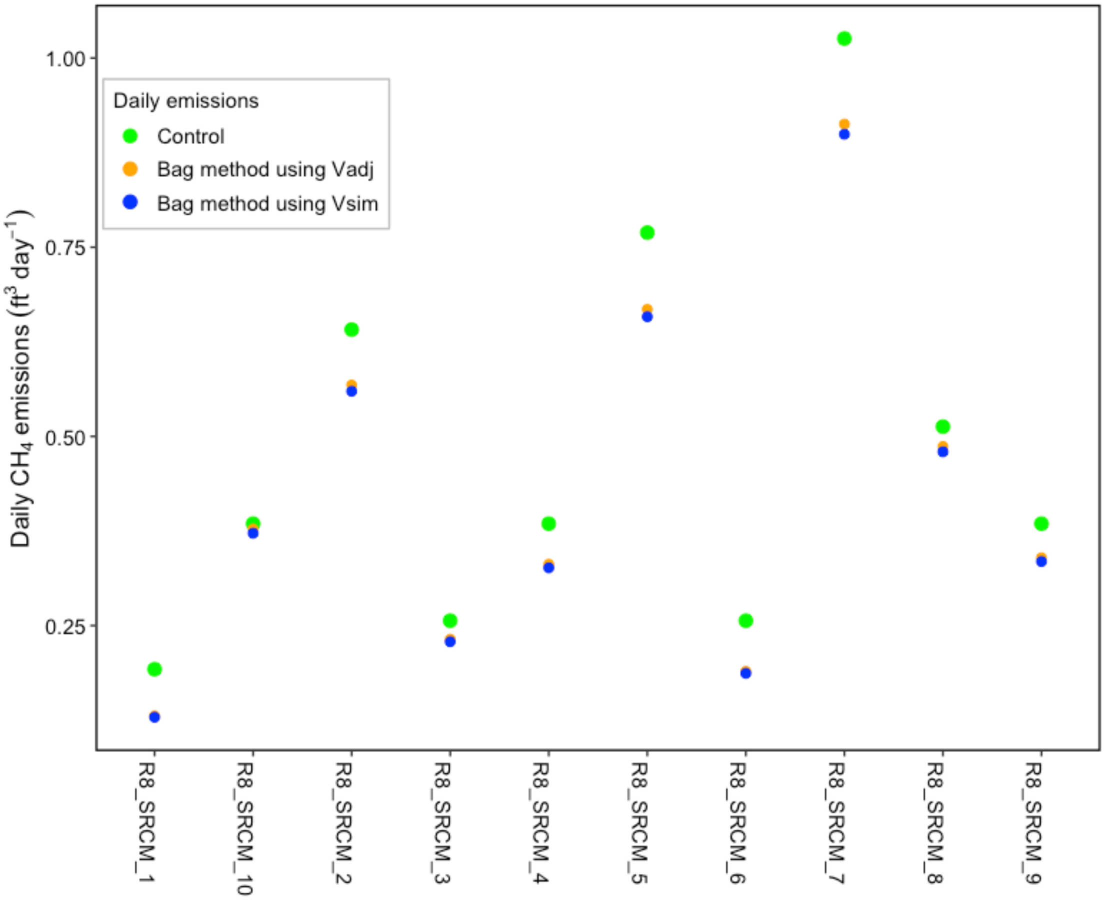

Figure 17. Bag method control tests results
