# Supplementary material for: A simple method to measure methane emissions from indoor gas leaks
Source: PLoS One. 2023 Nov 30;18(11):e0295055. doi: 10.1371/journal.pone.0295055 (PMC10688665; doi:10.1371/journal.pone.0295055)
Supplement: S7 Appendix — (PDF) [file pone.0295055.s007.pdf]

S7 Appendix: Leaks

S7 Appendix section 1: Leak locations by floor or location relative to natural gas meter

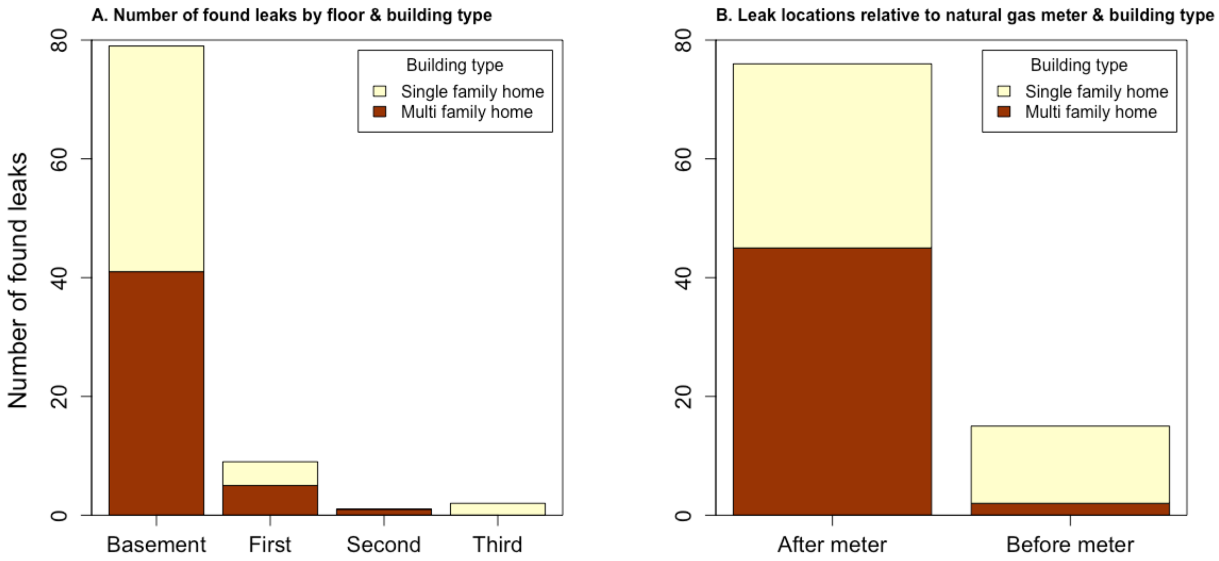

Figure 18. Leak locations

S7 Appendix section 2: Leak sources

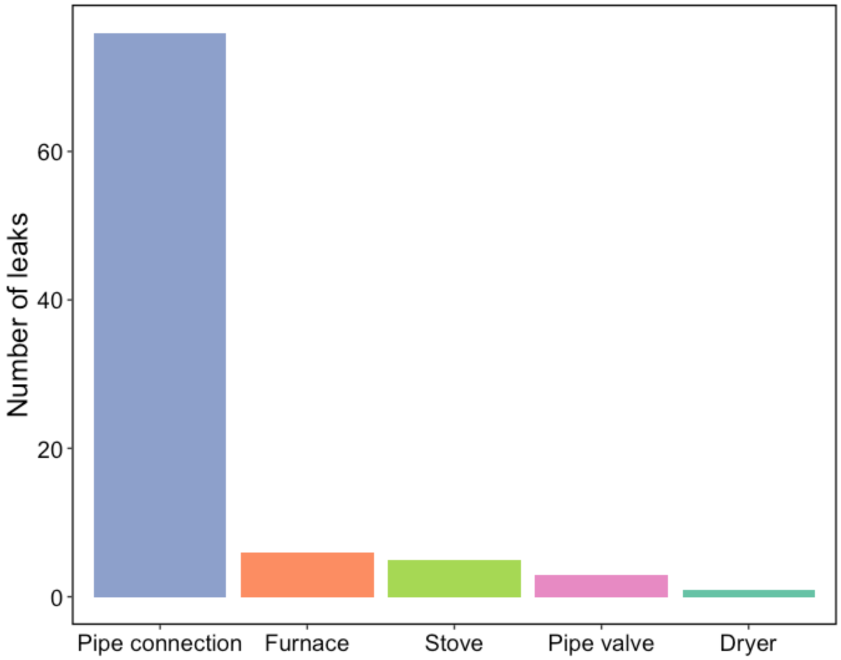

Figure 19. Leak sources
