# Supplementary material for: A simple method to measure methane emissions from indoor gas leaks
Source: PLoS One. 2023 Nov 30;18(11):e0295055. doi: 10.1371/journal.pone.0295055 (PMC10688665; doi:10.1371/journal.pone.0295055)
Supplement: S8 Appendix — (PDF) [file pone.0295055.s008.pdf]

S8 Appendix: Building age relationships

Building age didn't appear to predict the number of surveyed leaks found (R^2 = 0.088, n=13, p=0.326), RCM simple slope calculated flux (R^2 = 0.028, p=0.537, n=16), or the sum of leakage as quantified by the CGI (R^2=0.08, n=13, p=0.348).

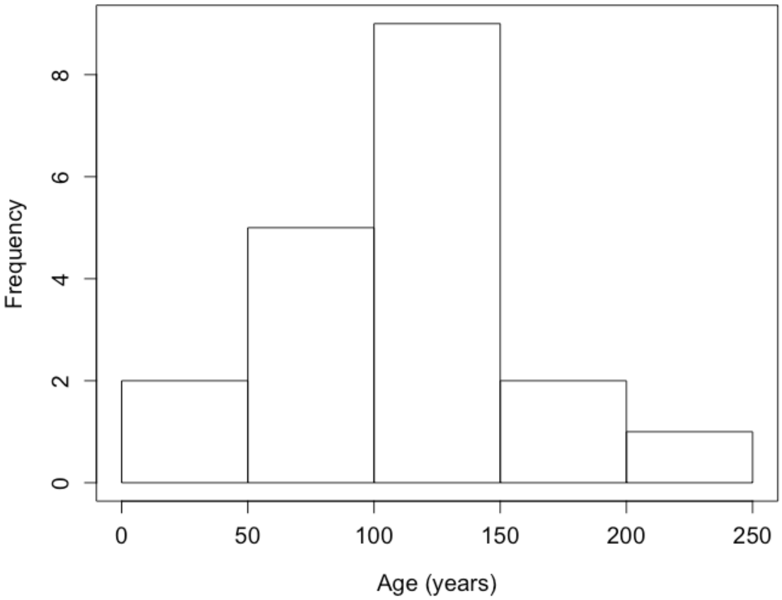

Figure 20. Histogram of ages of buildings studies (n=19)
