# Supplementary material for: A simple method to measure methane emissions from indoor gas leaks
Source: PLoS One. 2023 Nov 30;18(11):e0295055. doi: 10.1371/journal.pone.0295055 (PMC10688665; doi:10.1371/journal.pone.0295055)
Supplement: S9 Appendix — (PDF) [file pone.0295055.s009.pdf]

S9 Appendix: Instrument Quality Control Tests

S9 Appendix section 1: Picarro GasScouter G4301 Analyzer cavity ring-down spectrometer

We used a GasScouter for i) air CH_4 data logging during RCM and the bag method experiments ii) ambient air CH_4 concentration measurements on each floor of buildings and for outside baseline atmospheric measurements iii) analyzing the bag method air samples iv) metered control testing. The performance specification sheet from Picarro for the GasScouter is below.

Table 5. GasScouter performance specifications (copied directly from Picarro, Inc.)

Picarro G4301 GasScouter Performance Specifications			
Specification	CO_2	CH_4	H_2O
**Raw precision** (5 sec)	0.4 ppm + 0.1% of reading<br>Typical = **0.15 ppm***	3 ppb + 0.1% of reading<br>Typical = **0.8 ppb***	100 ppm + 5% of reading
**Precision** (300 sec, 1σ)	0.04 ppm + 0.02% of reading<br>Typical = **0.025 ppm***	0.3 ppb + 0.02% of reading<br>Typical = **0.1 ppb***	10 ppm + 5% of reading
**Lower Detection Limit** (300 sec, 3σ)	0.12 ppm<br>Typical = **0.075 ppm***	0.9 ppb<br>Typical = **0.3 ppb***	–
**Drift** (24 hr, peak-to-peak 50 min average)	0.5 ppm<br>Typical = **0.18 ppm***	1 ppb<br>Typical = **0.56 ppb***	–
**Measurement Range**	0–3%	0–800 ppm	0–3% (non-condensing)
**Measurement Interval**	3 sec		
**Response Time**<br>*(Rise/Fall Time 10–90%/90–10%)*	5 sec		

*Typical performance is defined as the median of testing results from 29 sequentially built G4301 analyzers measured at ambient concentration levels.

We measured an average data logging rate of 1.09 data points per second. To test the GasScouter, a Tedlar bag was fully evacuated and filled with a test gas. The bag was then attached to the Scout intake. We performed quality control tests using 3 different test gasses on September 17 2022. The GasScouter measured CH_4 concentrations to within 1.1% of test gasses as shown below.

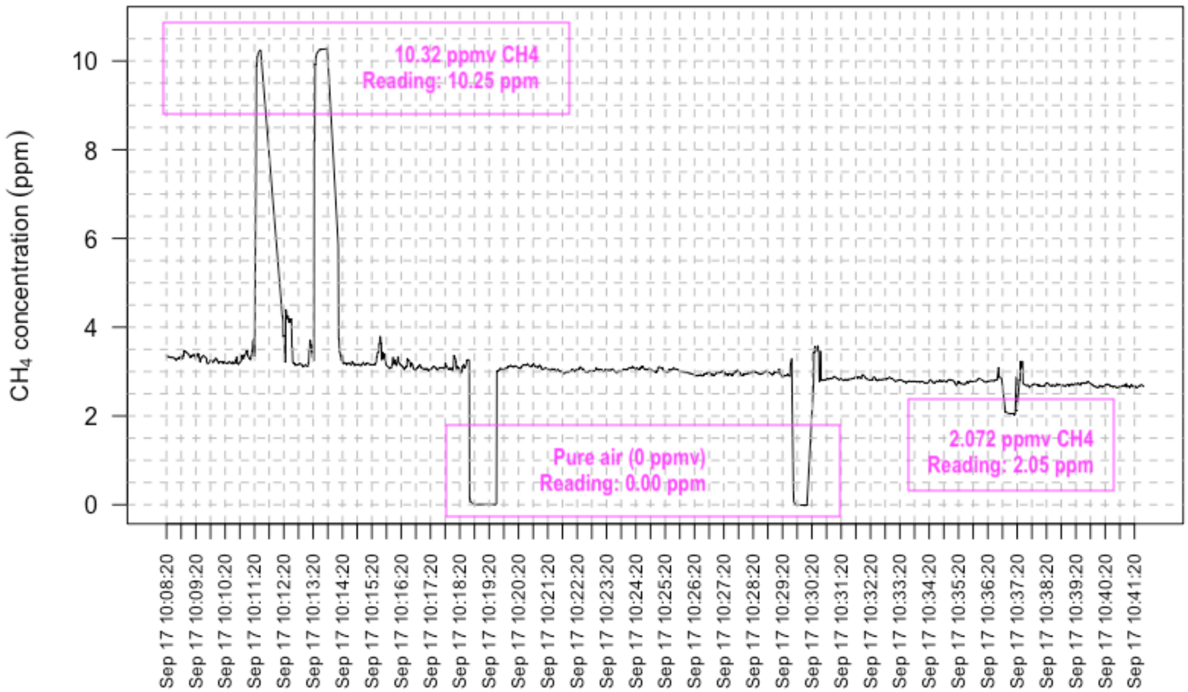

Figure 21. Testing the GasScouter

S9 Appendix section 2: Picarro G2311-f Cavity Ring-Down Spectrometer (CRDS)

We used a G2311-f for i) analyzing some the bag method air samples ii) metered control testing. The specification sheet from Picarro for the G2311-f is below.

Table 6. G2311-f performance specifications (copied directly from Picarro, Inc.)

G2311-*f* Performance Specifications	CO_2	CH_4	H_2O
**Flux Modes**			
3-Species Precision (10Hz, 1-σ)	≤200 ppb	≤3 ppb	≤6 ppm + 0.3% of reading
Max Drift at STP over 24 Hours/1 Month<br>**Max - Min of 50 minute average*	≤250/500 ppb	≤2.5/3 ppb	≤10 ppm + 0.3% of reading
2-Species Precision (10 Hz, 1-σ)	≤110 ppb	N/A	≤6 ppm + 0.3% of reading
Measurement Rate	≥10 Hz	≥10 Hz	≥10 Hz
Gas Response in Cell	≥5 Hz	≥5 Hz	≥5 Hz
**Precision Mode**			
Precision (1-σ of Raw, 5 sec/5 min averaged data)	≤150/50 ppb	≤1/0.7 ppb	≤6 ppm + 0.3% of reading
Max Drift at STP over 24 Hours/1 Month<br>**Max - Min of 50 minute average*	≤150/500 ppb	≤1/3 ppb	≤10 ppm + 0.3% of reading
Measurement Rate	≥0.2 Hz	≥0.2 Hz	0–3% (non-condensing)
Gas Response in Cell	≥0.33 Hz	≥0.33 Hz	≥0.33 Hz
**All Modes**			
Guaranteed Specifications Range	300–500 ppm	1–3 ppm	0–3 %v H_2O 25°C dew pt (non-condensing)
Operating Range	0–1000 ppm	0–20 ppm	0–7 %v H_2O 39°C dew pt (non-condensing)

We performed quality control tests using 3 different test gasses on September 17 2022. To test the G2311-f, a Tedlar bag was fully evacuated and filled with a test gas. The bag was then attached to the G2311-f intake. The G2311-f measured CH_4 concentrations to within 0.4% of test gasses as shown below.

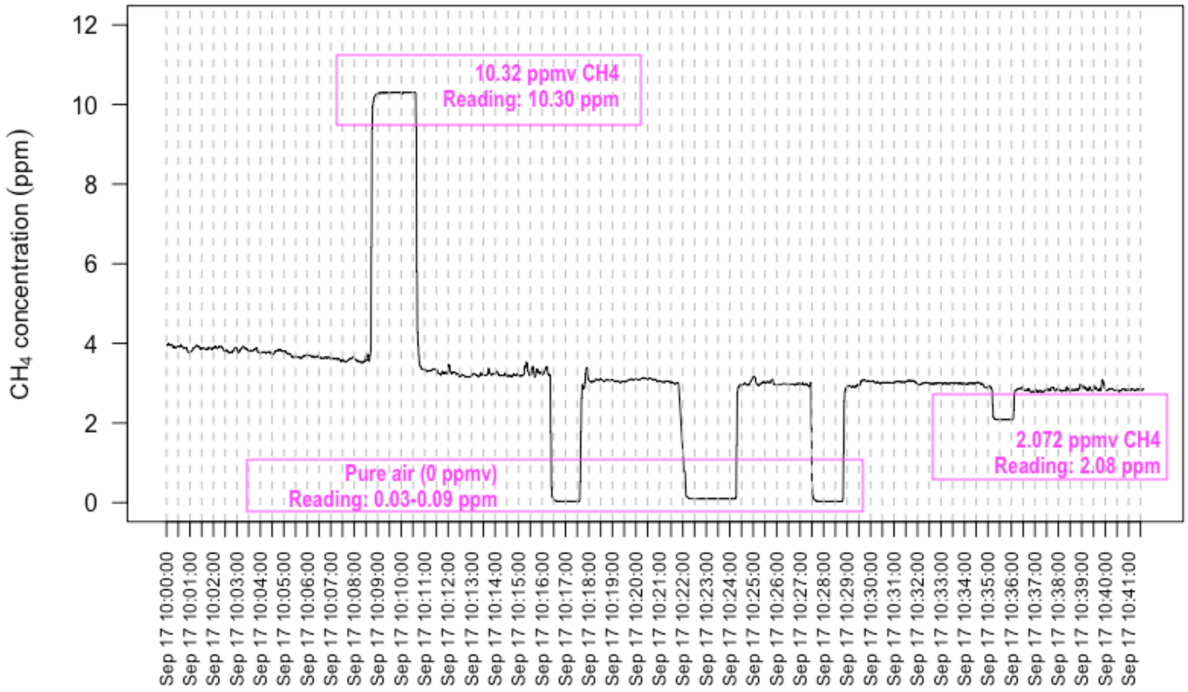

Figure 22. Testing the G2311-f

S9 Appendix section 3: Bascom-Turner Gas-Rover VG211 Portable Combustible Gas Indicator (CGI)

We used a Gas-Rover in Survey mode for indoor leak surveying. The Gas-Rover has the following CH_4-related specifications:

- CH_4 Sensors: Dual Catalytic Combustion
- Detection ranges: 0 to 10000 ppmv CH4 by volume, 0 to 100% CH4 by volume
- Accuracy of detection: 2% of reading +/- 20 ppmv; +/- 0.1% to 5%; +/-2.0% to 100 vol %
- Measurement resolution: 1 ppmv from 0 to 40000 ppmv; 0.05 vol % from 0 to 5 vol %; 1.0 vol % from 5 to 100 vol %

More details can be found here.

We calibrated the Gas-Rover using 2.5% CH_4 calibration gas from Bascom-Turner (part number MC-105) on Jul 13 2022, September 14 2022, September 19 2022 and on September 28 2022.
