# Supplementary material for: A simple method to measure methane emissions from indoor gas leaks
Source: PLoS One. 2023 Nov 30;18(11):e0295055. doi: 10.1371/journal.pone.0295055 (PMC10688665; doi:10.1371/journal.pone.0295055)
Supplement: S10 Appendix — (PDF) [file pone.0295055.s010.pdf]

S10 Appendix: Individual RCM experiment data and modeling plots

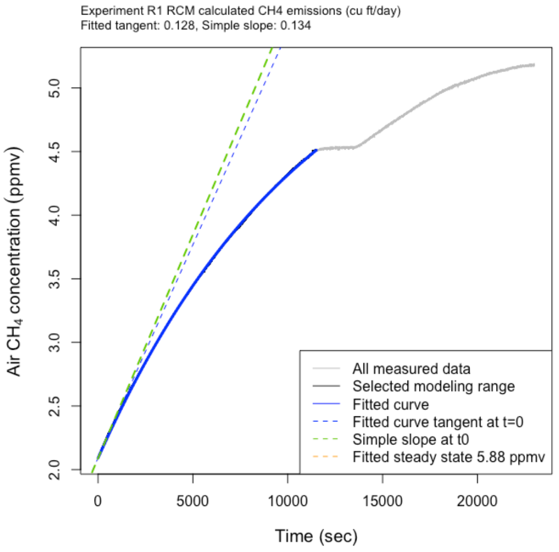

R1 note: fitted tangent using first 24000 sec was 0.132 ft^3 day^{-1}.

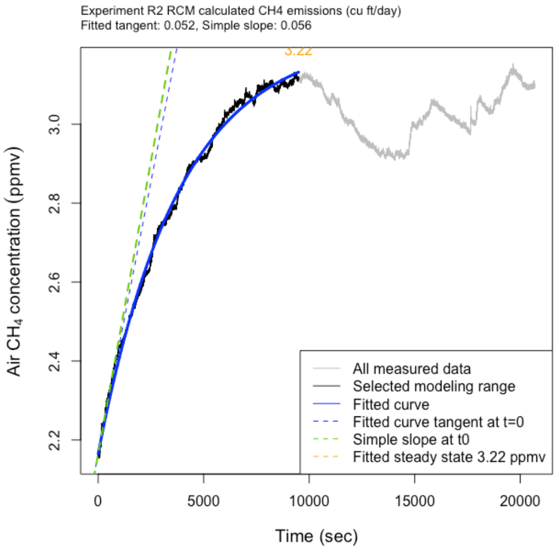

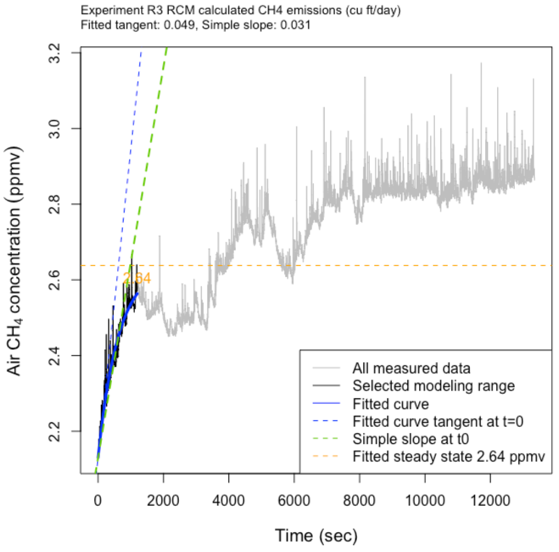

R3 note: fitting with 13000 sec results in a fitted tangent flux of 0.015 ft^3 day^{-1}

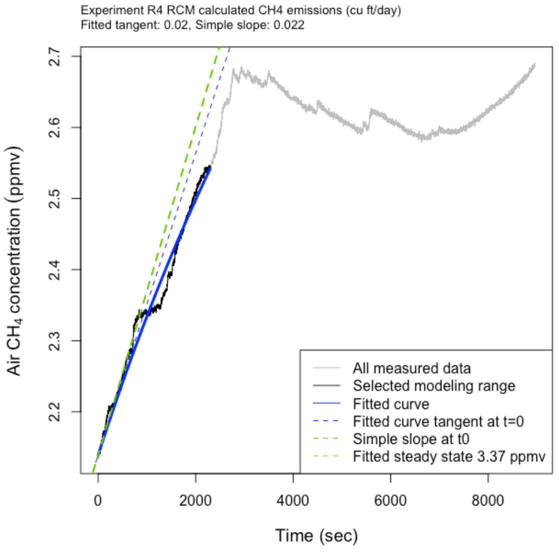

R4 note: fitting with 9000 sec results in a fitted tangent flux of 0.033 ft^3 day^{-1}.

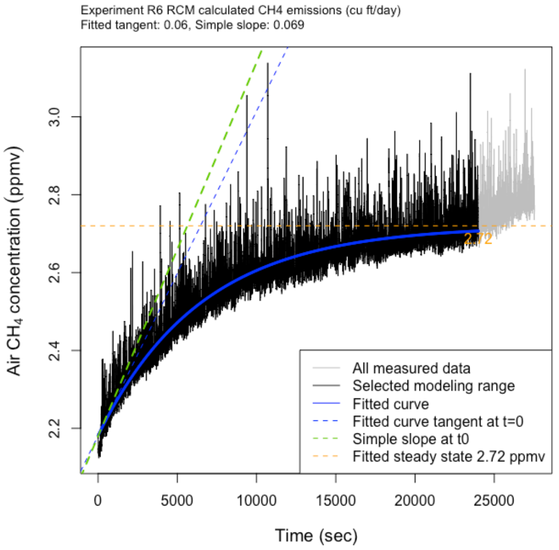

R6 note: this experiment was repeated - see SI 4.8 for more details.

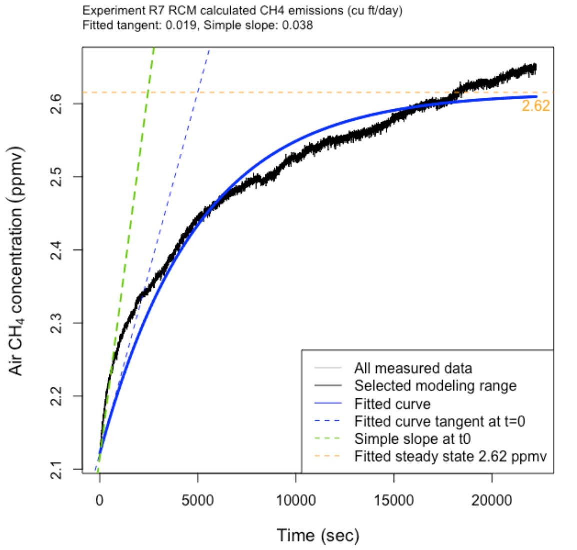

R7 note: this experiment was a repeat of R6 - see SI 4.8 for more details.

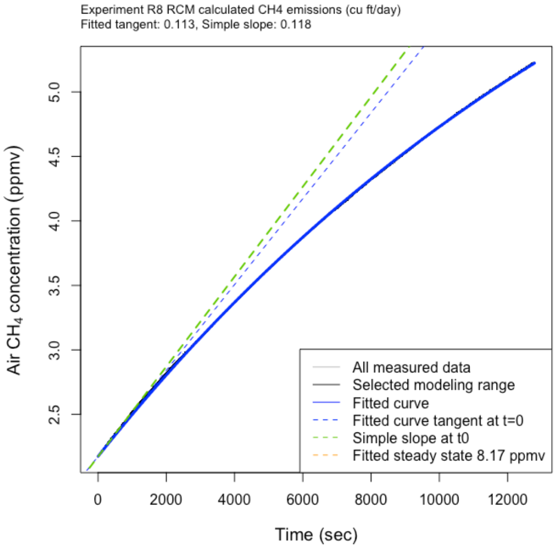

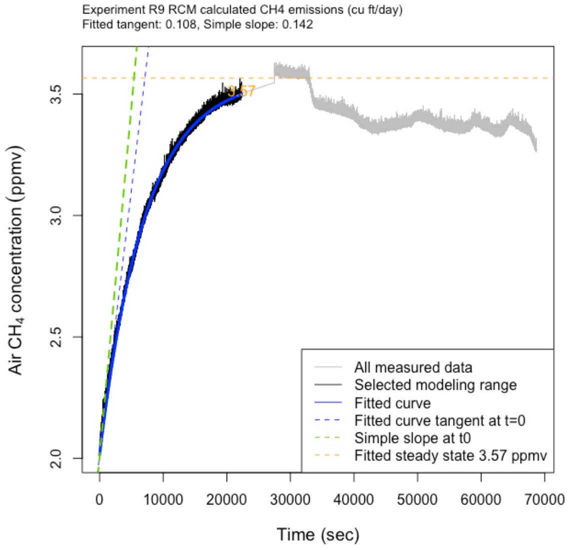

R9 note: a power outage resulted in no data between approximately 22000 to 26000 sec

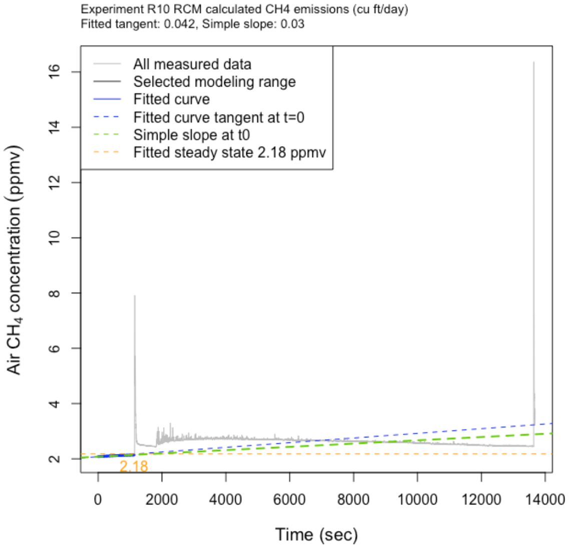

R10 notes: (i) spikes were caused by unburned methane emanating from domestic water NG boiler injectors upwind of the GasScouter sensor. See also section SI 4.6. (ii) fitted tangent using first 4000 sec was 0.19 $ft^3\ day^{-1}$

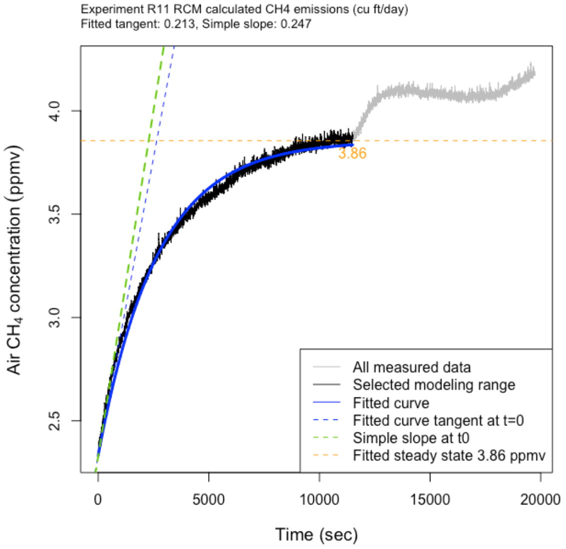

R11 note: fitting with 20000 sec results in a fitted tangent flux of 0.17 $ft^3\ day^{-1}$.

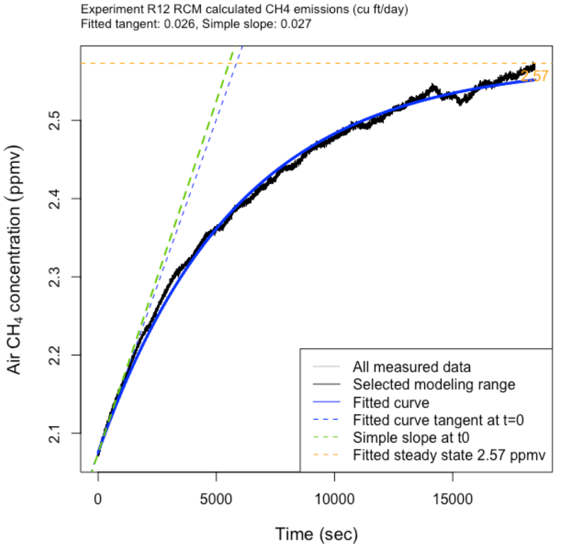

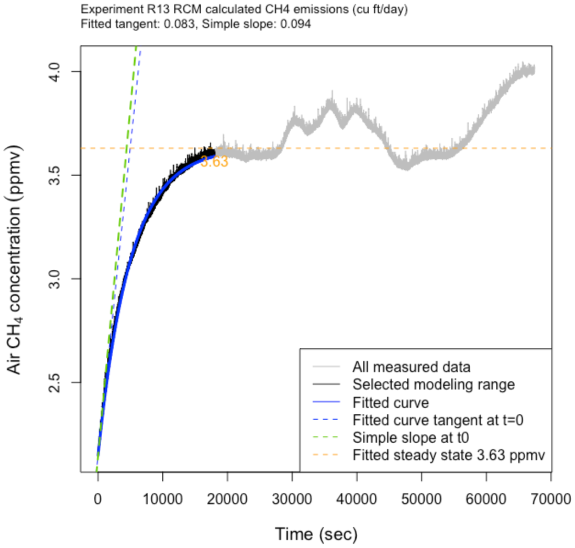

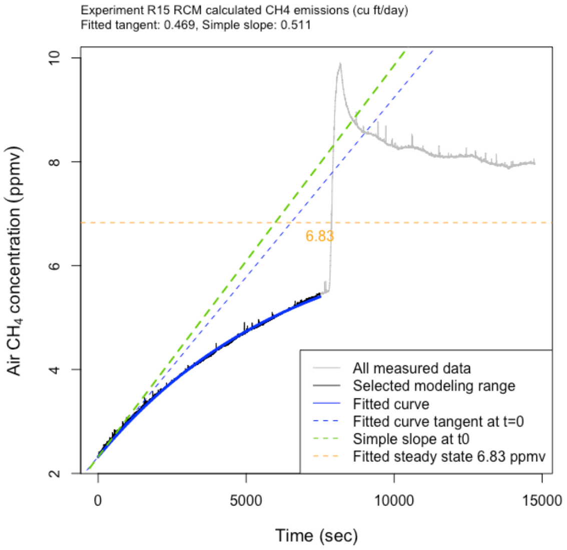

R15 notes: (i) spike example - see section SI 4.6 (ii) fitted tangent using first 15000 sec was 0.49 ft^3 day^{-1}

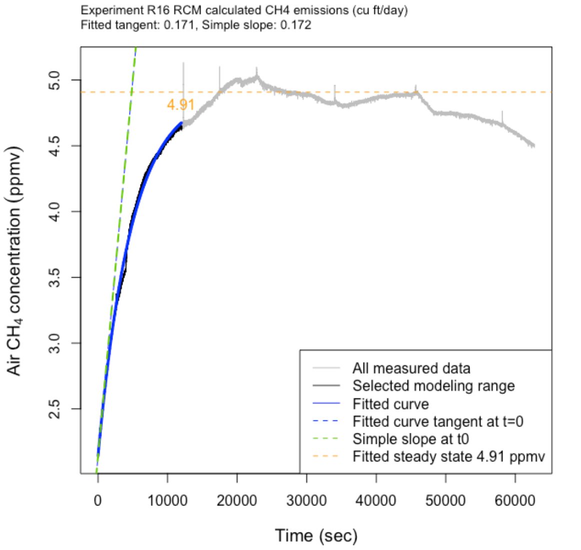

R16 notes: (i) spike example - see section SI 4.6 (ii) fitted tangent using first 25000 sec was 0.16 ft^3 day^{-1}

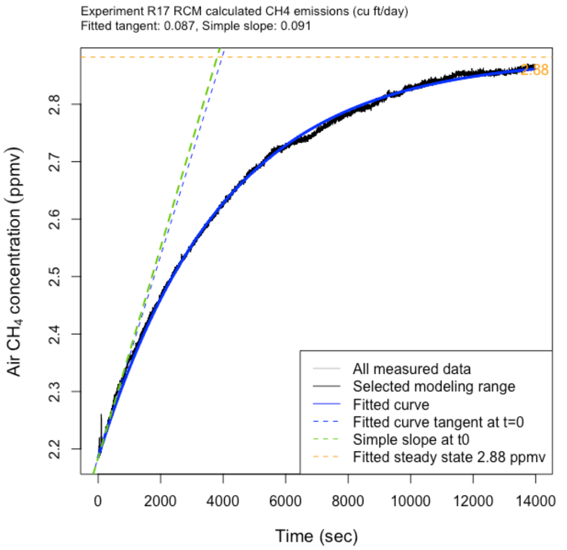

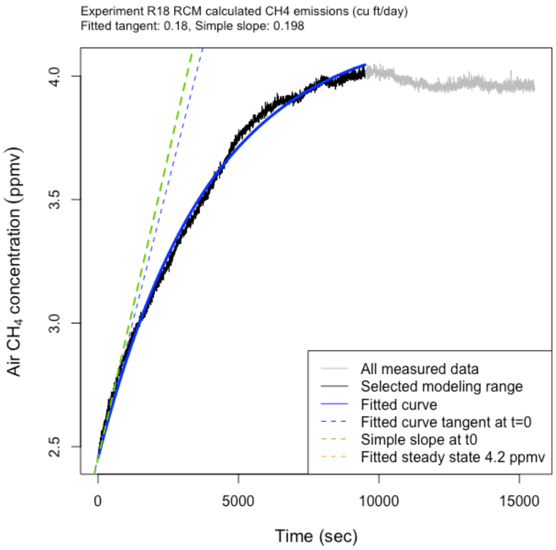

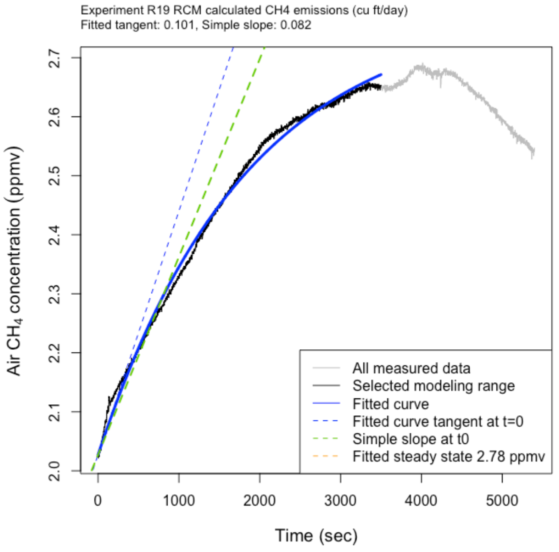

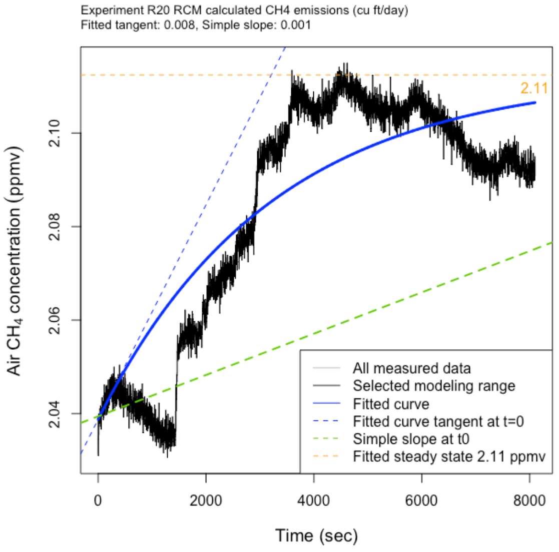

R20 note: using data between 1600 and 4250 sec results in a fitted tangent flux of 0.01 ft^3 day^{-1}

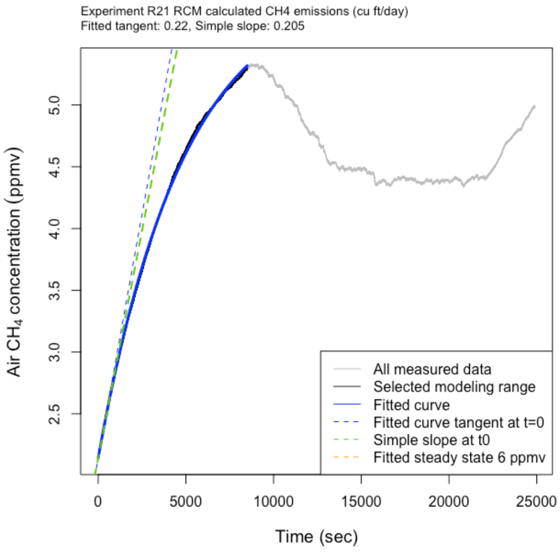

Figure 23. Room chamber method (RCM) data and modeling plots
